# Fluxion Potencies

**Published:** 1882-04

**Authors:** Thos. Skinner

**Affiliations:** 25 Somerset Street, Portman Square, London, W.


### FLUXION POTENCIES.

*Editor of* THE HOMŒOPATHIC PHYSICIAN:

SIR: If it is not too late, I should like to be allowed to make a correction in Dr. W. J. Guernsey's paper in the number of your journal for October, 1881 (Vol. I). I was travelling at the time, and I only saw the number for the first time lately. Dr. Guernsey quotes from *The Organon*, Vol. III, p. 327, that I have stated that "Hahnemann's MM can be made by his (Dr. Skinner's? ) fluxion attenuator, in 3d., 2h. and 4½m."

As many of your readers might easily get confused between Dr. Fincke's original process of attenuation by means of fluxion, that is *continuous* fluxion, and my own by *interrupted* fluxion, permit me to say that the time above given refers to Dr. Fincke's continuous-process. After the words "fluxion attenuator," Dr. Guernsey will find

*Though we cannot indorse all that Prof. Butlerow advances in his letter, we publish it in full at the request of Dr. Fellger.—Editor H. P.

the words "on the principle of Dr. Fincke;" and at the end of the paragraph he will find it distinctly stated, "interrupted fluxion requires 14 days!"

In conclusion, I am still perfectly satisfied that Dr. Fincke's process is the best, and certainly the quickest and most economical. It simply requires a changed notation, such as I have given in *The Organon*, Vol. III.

THOS. SKINNER, M. D.,
25 Somerset Street, Portman Square, London, W.

*March* 1, 1882.

